# Hoxc10‐mediated ‘positional memory’ regulates cartilage formation subsequent to femoral heterotopic grafting

**DOI:** 10.1111/jcmm.70140

**Published:** 2024-10-21

**Authors:** Haoyue Song, Yujia Hao, Qingpeng Xie, Xiaohang Chen, Na Li, Jia Wang, Xiaoxuan Zhang, Yuan Zhang, Jinjia Hong, Shuyun Xue, Pengfei Zhang, Si Xie, Xing Wang

**Affiliations:** ^1^ Shanxi Medical University School and Hospital of Stomatology Taiyuan China; ^2^ Shanxi Province Key Laboratory of Oral Diseases Prevention and New Materials Taiyuan China

**Keywords:** bone healing, bone marrow mesenchymal stem cells, cross‐layer bone graft, endochondral ossification, Homeobox c10 (Hoxc10)

## Abstract

The Hox gene plays a crucial role in the bone development, determining their structure and morphology. Limb bone grafts expressing Hox positive genes are commonly used for free transplantation to repair Hox negative mandibular critical bone defects. However, the specific role of original Hox genes in newly formed bone during the cross‐layer bone grafting healing process remains unexplored. Our findings demonstrate that femurs ectopically grafted into the mandibular environment retained a significant ability to differentiate into cartilage and form cartilaginous callus, which may be a key factor contributing to differences in bone graft healing. Hoxc10, an embryonic layer‐specific genes, regulates cartilage formation during bone healing. Mechanistically, we observed Hoxc10 retention in co‐cultured femoral BMSCs. Knocking out Hoxc10 narrows the bone gap and reduces cartilage formation. In summary, we reveal Hoxc10's ‘positional memory’ after adult cross‐layer bone graft, influencing the outcomes of autologous bone graft.

## 1 INTRODUCTION

Until now, autogenous bone grafting remains an indispensable procedure for achieving optimal restoration of critical bone defects.^1^, ^2^ The survival and long‐term protection of transplanted bone are influenced by various factors, among which selecting the appropriate donor site has a decisive impact on the entire process.^3^, ^4^ Research has shown that the efficacy of autologous mandible repair surpasses that of limb bones: the resorption rate of bone blocks after 1 year for autologous appendicular skeleton graft repair in mandibular defects ranges from 25.8% to 41.8%, whereas the resorption rate observed in mandible defects repaired using autologous craniofacial bone block grafts is merely between 8.4% and 19.8%.^5^, ^6^ This indicates that the therapeutic effect of autologous mandibular bone repair is superior to that of limb bone repair, which may be due to the formation of cartilage in the progress of limb bone heterotopic grafts.^7^ The specific manifestation is that when the mandibular periosteum is peeled off and grafted to the tibial defect, the same osteogenic effect as in the homotopic graft is observed; however, when the tibial periosteum was grafted into the mandibular defect, it was cartilage rather than the bone found in the injured area.^7^ It is currently unknown whether there are other cells or tissues in the limb bones and mandible that affect the therapeutic effect of heterotopic graft, in addition to the periosteum. Considering that autologous appendicular skeleton grafts are still the gold standard in the clinical treatment of large‐scale mandibular defects,^8^ further analysis is needed to determine the reasons for the cartilage formation during autologous appendicular skeleton grafted to the mandible, to optimize bone healing during the transplantation process.

The Homeobox (Hox) family genes naturally become a key regulator during the process of bone graft and repair.^9^, ^10^, ^11^, ^12^ Hox gene is a class of major regulatory genes that control development and cell differentiation, and is an important marker reflecting the origin of the embryonic origins in various parts of the body.^11^ During embryonic development, differences in Hox expression give spatial specificity to cells in different parts of the front and back axes of the body, ensuring that organisms develop normal forms of trunk, limbs, skull and other organs at predetermined locations.^13^ Limb bones and mandibles come from the mesoderm and neural crest, respectively, which leads to differences in their osteogenic differentiation patterns and gene expression profiles.^14^, ^15^ Limb bones are ossified through endochondral ossification where marrow mesenchymal stem cells (MSCs) first become chondrocytes, then differentiate, hypertrophy, at the meantime, vascular ingrowth, bone matrix replace the initial cartilage and ultimately form a fully developed bone.^16^ In contrast, maxillofacial bone originating from the neural crest directly induces marrow‐derived MSCs to become osteoblasts, forming stable woven bone without an intermediate cartilage stage.^17^, ^18^ Therefore, the presence or absence of cartilage replacement is a crucial transitional stage between intramembranous ossification and endochondral ossification. In addition, RNA sequencing results showed that MSCs in the maxilla and mandible were Hox negative, while MSCs in the iliac bone were positive.^19^ However, the reason for the formation of cartilage in the mandible after femoral ectopic transplantation is currently unclear in the graft environment. In addition, whether Hox genes of femoral grafts retain memory in the new mandible environment and how Hox genes affect bone formation in the process of graft have never been involved at present, which is difficult to explain by simple differences in embryonic‐related genes.

To investigate the differences in healing outcomes after graft of different autogenous bone grafts into the mandible, we established three groups of autograft models: femoral homotopic grafts, femoral heterotopic, and mandibular homotopic grafts. By analysing tissue staining in the three groups, we focused on cartilage expression in the femur and mandible transplanted into the mandible, respectively. To further investigate which gene plays a role in mandible repair after graft, three groups of post‐graft osteochondral tissues were removed and subjected to RNA sequencing, suggesting that the cartilage appearing in mandible regeneration may be related to Hoxc10. Notably, Hoxc10 in femoral implant blocks did not show significant differences before and after graft, and showed significant ‘positional memory’ throughout the bone healing and regeneration process. In vitro, we simulated the graft environment and investigated the differentiation potential of femoral bone marrow mesenchymal stem cells (BMSCs). We examined the expression levels of cartilage‐related genes and Hoxc10 in femoral BMSCs transplanted into the environment of mandibular BMSCs. In vivo, we interfered with Hoxc10 after performing femoral heterotopic graft to observe cartilage formation. In summary, we identified an important role for Hoxc10 in autologous bone graft healing and demonstrated in vivo the positive role of regulating this factor in autologous bone grafting, with potential implications for clinical and tissue engineering.

## 2 METHOD AND MATERIALS

### 2.1 Construction of animal model of autogenous bone grafting

All animal experiments in this study were approved by the Ethics Committee of the Shanxi Medical University (Approval ID: 2022–066). A total of 40 male SD rats were housed in the specific pathogen‐free (SPF) laboratory conditions at the animal experimental center of Shanxi Medical University. Anaesthesia was induced using pentobarbital sodium (40 mg/kg). Femoral or mandibular blocks were harvested using a 3 mm diameter annular bone drill. The rats were randomly assigned to three grafting groups: femoral homotopic grafting, mandible homotopic grafting, and femoral heterotopic grafting. All experimental animals were euthanized after 6 weeks. The procedures were performed according to the ethical approval guidelines of Stomatological Hospital of Shanxi Medical University for animal studies (2022‐066).

### 2.2 Raman spectrum assay

We euthanized the rats 6 weeks after femoral homotopic grafting, mandible homotopic grafting, and femoral heterotopic grafting, dissected the mandible and femur completely. We could observe the transplantation site (with a 3 mm diameter circular bone drill mark on the mandible or femur), and separated the surrounding 1 × 1 cm as the sample analysis area for the transplanted bone. The femur or mandible was soaked in glycerol for transparency and removed every 10 min, with a soaking time of 60 min. Detection of newly formed bone fragments at bone sutures after transplantation using a Raman system (inVia, Renishaw, UK) with an excitation wavelength of 785 nm. The scanning spectrum range was 300–1800 cm^−1^, and the resolution was 0.5 cm^−1^. The technical support of spectral acquisition, calibration, and preprocessing for the bone spectra was provided by the Institute of Laser Spectroscopy Shanxi University. The Mineral to Matric ratio of phosphate to amide III (962/1280 cm^−1^) reflects the mineralization degree of bone tissue. The 1063/958 cm^−1^ ratio served as a spectral indicator for evaluating the relative content of cartilage and bone. After minimizing spectral noise, smoothing the process and normalizing Raman spectra, we calculated the mineral‐to‐matrix ratio of region of interest of newly formed bone.

### 2.3 Histomorphology

The newly formed bone fragments at bone sutures were carefully selected and subsequently sliced into continuous sections. The paraffin slices were stained with haematoxylin and eosin, Goldner, Safranin O/fast green and Masson after conventional dewaxing to water. And they were analysed by using the Osteomeasure and Aperio Image Scope image analysis systems (OsteoMetrics, Atlanta, GA, USA).

### 2.4 Immunofluorescence

The sections underwent epitope recovery by treatment with EDTA Antigen Retrieval Solution (AR0023, BOSTER, Wuhan, China). Subsequently, the sections were pretreated with a blocking solution containing normal goat serum at 37°C for 30 min and then stained using primary antibodies. The primary antibodies used in this study included Rabbit‐anti‐Hoxc10 (A09018‐1, BOSTER) (1:100), Rabbit‐anti‐Sox9 (A00177‐2, BOSTER) (1:50), Mouse‐anti‐CD105 antibody (ab230925, Abcam), (1:100) and mouse‐anti‐CD44 (M00052‐3, BOSTER) (1:100). Following an overnight incubation at 4°C, the sections were rinsed with PBS and subsequently incubated with secondary anti‐rabbit fluorescent antibodies (Dylight 594) (BA1142, BOSTER) (1:500), secondary anti‐mouse fluorescent antibodies (Dylight 488) (BA1126, BOSTER) (1:500), secondary anti‐rabbit fluorescent antibodies (Dylight 488) (BA1146), secondary anti‐mouse fluorescent antibodies (ab150116, Abcam) (1:500) and DAPI.

### 2.5 Immunohistochemical study

Immunohistochemical studies according to standard protocols. After slicing the transplanted bone tissue, dry it in a 60°C oven for 2 h to complete the specimen preparation, followed by dewaxing and hydration. Use antigen repair solution to perform high‐temperature repair on the slices for 10 min to restore the antigen epitopes hidden due to antigen blockade or denaturation. Seal after incubation with hydrogen peroxide. Subsequently, the primary antibody (CD31, Abcam, ab182981, 1:100; CD10a1, BOSTER, BA0533, 1:100) was incubated overnight at 4°C. The HRP labelled secondary antibody was diluted 100 times with PBS and dropped onto slices completely covering the tissue. Incubate at 37°C for 60 min in a wet box. Use 3,3′‐diaminobenzidine (DAB) for colour development, lightly counterstain the cell nucleus with haematoxylin, seal with neutral gum, and observe under a light microscope.

### 2.6 RNA sequencing

RNA sequencing was performed by Beijing Genomic Institute (BGI, Shenzhen, China). Separate the 1 × 1 cm bone around the transplant site, freeze it in liquid nitrogen, and place it in a mortar. Continue to maintain a low temperature environment for the sample with liquid nitrogen. Grind the tissue into powder using a frozen pestle to ensure complete fragmentation. Subsequently, the ground bone tissue was rapidly transferred to 1.5 mL of TRIzol lysis buffer and incubated for 5 min. After mRNA isolation, fragmentation, cDNA synthesis, end repair, addition of A and adaptor ligation, PCR amplification and library quality control steps were carried out. High‐quality DNA nanoballs were then loaded onto patterned nanoarrays using high‐intensity DNA nanochip technique and subjected to combinatorial Probe‐Anchor Synthesis for sequencing analysis. Finally, Dr. Tom online software developed by BGI was employed to analyse the interaction network involving gene interactions, co‐expression patterns and regulatory mechanisms among these genes in a comprehensive manner. (http://report.bgi.com/).

### 2.7 Isolation and culture of L‐BMSCs and M‐BMSCs

Primary BMSCs were established by tissue mass culture from the limb bone (L‐BMSCs) or mandible (M‐BMSCs) of the 4‐week‐old male SD rats. Rats were euthanized, and the skin was cut open to expose the mandible and bilateral femurs. The femurs were dissected from both hip sockets and knee joints, and the mandible was dissected from both condylar processes. The muscle tissue was then soaked in PBS containing 1% bispecific antibody. L‐BMSCs: Wear sterile gloves, use scissors and tweezers to remove clean muscles, and then use bone biting forceps to remove both ends of the femoral metaphysis, exposing the bone marrow cavity. Use a 1 mL syringe to aspirate PBS and flush out the bone marrow into an EP tube, 1000 rpm/min, centrifuge for 5 min, discard the supernatant and resuspend in 10% culture medium into a culture bottle (Corning, USA). Jawbone group (M‐BMSCs): Muscle tissue was also removed using sterile scissors, and mandibular bone marrow suspension and shredded mandibular tissue were cultured together in 5 mL of 15% culture medium. The culture medium was changed every 2 days. All operations are carried out in a sterilized biosafety cabinet (Thermo, Germany).

### 2.8 Transwell model construction

The coculture systems were established using 6‐well Transwell plates with a pore size of 0.4 μm (NEST, China). Cells were seeded in the lower chambers at a density of 2.0 × 10^5^ cells/well and allowed to attach firmly to the wall for 24 h. Subsequently, the upper chambers containing 4.0 × 10^4^ cells/well were transferred to the lower plates, thereby establishing a co‐culture system. L/L BMSCs group and M/M BMSCs means L‐BMSCs and M‐BMSCs are seeded both in the upper and lower compartments, which simulates the in vivo experimental femoral homotopic grafting and mandible homotopic grafting, respectively. L/M‐BMSCs group are seeded with L‐BMSCs in the upper layer and M‐BMSCs in the lower layer, which simulates the in vivo experimental femoral heterotopic graft group. The plates were incubated at 37°C in a CO_2_ incubator for 7 days. Following this, the lower chambers of Transwell plates were relieved and all cells located in upper chambers underwent subsequent analysis including quantitative real‐time PCR (qRT‐PCR).

### 2.9 Alcian blue staining

Every 3 × 10^5^ target BMSCs were transferred to 15 mL centrifuge tube. After centrifugation, BMSCs were exposed to chondrogenic medium according to the protocol of Sprague–Dawley Rat Bone Marrow Mesenchymal Stem Cells Chondrogenic Differentiation Kit (HyCyte, BMRS‐D203, Suzhou, China). The chondrogenic medium was refreshed every 2 days. After 3 weeks of regular cell culture, Alcian blue staining was used to detect the formation of cartilage nodules.

Cartilage nodules were embedded in paraffin after 4% Paraformaldehyde (BOSTER, Wuhan, China) fixation, dehydration, xylene transparent. Slice the embedded sample to a thickness of 3–5 μm and place it on a glass slide. After placing the glass slides in Alcian blue solution (BOSTER, Wuhan, China) at room temperature for 30 min, dehydrate the slide three times (5 min each time) and cleared in xylene for 5 min, and then seal with neutral resin.

### 2.10 Transfection

The adenovirus particles expressing short hairpin (sh) RNAs interfering and overexpression Hoxc10 vector were designed by Hanbio Biotechnology Co., Ltd. (Hanbio Biotechnology, Shanghai, China). All the adenovirus particles expressed enhanced green fluorescent protein (GFP). As described by the manufacturer, M‐BMSCs or L‐BMSCs were transfected with the adenovirus particles in 6‐well plates to achieve the regulation of Hoxc10 expression level. When the cell fusion rate reaches 50%, add the adenovirus (Multiplicity of Infection, MOI = 100) to 250uL fresh complete culture medium per well according to the 1/2 volume method. After 4 h of infection, replenish complete culture medium to 2 mL per well. After 24 h of infection, the original culture medium was removed. After 48 h, the transfection efficiency of overexpression and knockout was verified through microscopy and q‐PCR experiments.

### 2.11 ChIP

Cells were cross‐linked with 1% formaldehyde at room temperature for 10 min, and the reaction was stopped by adding an appropriate amount of 10 × glycine and incubating at room temperature for 5 min. The cells were lysed, and the chromatin was treated with ultrasound (cut into 200–500 bp fragments). Five microgram of anti‐Hoxc10 antibody was used (Abcam, ab153904) was rotated overnight at 4°C to immunoprecipitate the lysate. After extensive washing, the chromatin was eluted and purified by reverse crosslinking at 65°C overnight. Purification of DNA using phenol chloroform extraction and ethanol precipitation. PCR amplification was performed using Sox9 gene promoter specific primers (Table 1). PCR products were analysed on 2% agarose gel in 1× TAE buffer at 100 V for 40 min. The gel was stained with ethidium bromide and observed under ultraviolet light. A unique band corresponding to the Sox9 amplicon was observed.

**TABLE 1:** The primer sequences used for RT‐qPCR and ChIP.

Primer	Sequence (5′ to 3′)
Sox9‐F	TGGCAGAGGGTGGCAGACAG
Sox9‐R	CGTTGGGCGGCAGGTATTGG
ChIP‐Sox9‐1‐203‐F ChIP‐Sox9‐1‐203‐R ChIP‐Sox9‐2‐225‐F ChIP‐Sox9‐2‐225‐R	ATACCTTCATCCCAGAGCACAG CAGTAAGCTACGACTCGGTTCG CTGTCTTCGCTAGAACACAGG GAGCAAAGTTGGATAAGGGAGG
Col2A‐F	GGAGCAGCAAGAGCAAGGAGAAG
Col2A‐R	GGAGCCCTCAGTGGACAGTAGAC
Hoxc10‐F	CAACACCTACCCGTCCTACCTCTC
Hoxc10‐R	GCAGCAGACATTCTCCTCCTTGAC
GAPDH‐F	ACGGCAAGTTCAACGGCACAG
GAPDH‐R	CGACATACTCAGCACCAGCATCAC

### 2.12 Bioluminescence imaging

Two groups of SD rats were anaesthetised with pentobarbital sodium, and then underwent femoral heterotopic graft surgery according to the aforementioned method. In order to verify the effect of Hoxc10 during bone graft, Adenovirus (AD)‐Hoxc10‐shRNA was synthesized by Hanbio (Hanbio Biotechnology Co., Ltd., Shanghai, China) and injected the virus locally between the femur grafts and the mandibles at a dose of 2 × 10^10^PFU/rat during femoral heterotopic graft. Another group was given AD‐NC (Hanbio Biotechnology Co., Ltd., Shanghai, China) in the same way. Four days later, the expression of adenovirus was detected by bioluminescence and photographic images. Sacrifice all rats at 6 weeks after surgery and remove bone tissue from the transplant site for further analysis.

### 2.13 Real‐time quantitative polymerase chain reaction (RT‐qPCR)

Total cellular RNA was isolated by Trizol (Mei5bio, Beijing, China). RNA concentrations and quality were verified by the Nanodrop 1000 spectrophotometer (Thermo Fisher Scientific, USA). First‐strand cDNAs were synthesized by using the ReverTra Ace qPCR RT kit (FSQ‐101, TOYOBO, OSAKA, Japan) according to the manufacturer's instructions. Following primers were synthesized from Sangon (Sangon, Shanghai, China):

The PCR reactions were performed using the 2X M5 HiPer Realtime PCR Super mix with Low Rox (MF797, Mei5bio, Beijing, China) on the Applied Biosystems QuantStudio Real‐Time PCR System (Applied Biosystems, USA). Specific conditions were as follows: initial denaturation 95°C for 15 s, followed by 40 cycles of 95°C for 15 s, 60°C for 15 s and 72°C for 30s. The relative gene expressions were assessed through the comparative threshold cycle (ΔΔCt) method using GAPDH as reference control.

### 2.14 Statistical analyses

The data was analysed using GraphPad Prism (Graph‐Pad Prism, San Diego, CA, USA). There were at least three biological and technical replicates performed for each sample. All data were expressed by mean ± standard deviation. The experimental data were compared by AVONA method and *t*‐test. The significance level of statistical test was set to *p < 0.05, **p < 0.01 and ***p < 0.001.

## 3 RESULTS

### 3.1 Variations in bone healing phenotypes observed between homotopic and heterotopic grafting procedures

**FIGURE 1:**
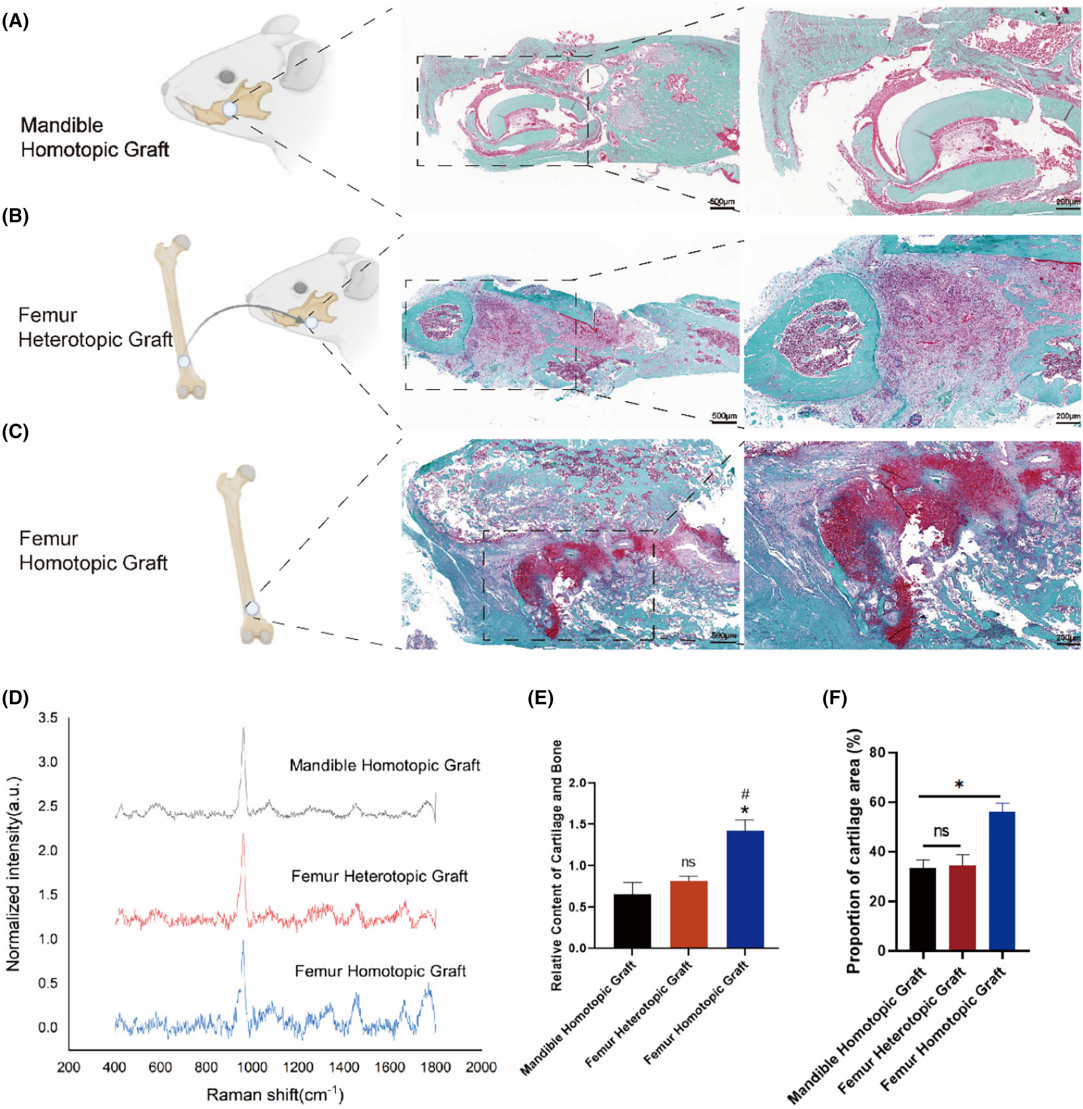
Safranin O/fast green staining and Raman spectroscopy of homotopic and heterotopic grafting has more cartilage compared to other groups. (A‐C) depicts the schematic diagrams of femoral homotopic grafts, femoral heterotopic grafts, and mandible homotopic grafts, respectively and the corresponding representative Safranin O/fast green staining image (*n* = 6). The image on the left represents the lower magnification (Scale bar = 500 μm), high magnification on the right. (Scale bar = 200 μm). (D)Representative Raman spectra of mandible homotopic graft, femoral heterotopic graft and femoral homotopic grafts were obtained for analysis. (E) Quantitative comparison of the area ratio of 1063/958 cm^−1^. (F) Quantitative analysis of red cartilage tissue in Safranin O/fast green staining image. The data are presented as the mean ± SD (*n* = 6). *p < 0.05.

We conducted bone graft procedures on Sprague–Dawley rats to investigate the regenerative mechanisms involved in mandibular defect repair following grafting of mesoderm limb bones and neural crest‐derived mandibular grafts, specifically focusing on the utilization of intramembranous ossification or endochondral ossification for bone and cartilage formation. To assess the grafts' contribution to bone regeneration within the donor environment and elucidate the underlying mechanism of intramembranous or endochondral ossification post‐grafting, we designated the femur and mandible as our control group (Figure 1A,C). In addition, we conducted femur heterotopic bone grafts as the experimental group, where mesoderm‐derived femurs were grafted into a mandible environment derived from neural crest cells (Figure 1B). Considering that 4–6 weeks of bone repair can be used as the dividing point of bone tissue towards intramembrane or endochondral ossification, we removed bone callus from the graft area at 6 weeks after grafting. The Safranin O/fast green staining results indicate that the bone injury repair mechanism was activated in the aforementioned cases, leading to successful survival of bone grafts and partial reconstruction of bone sutures through regenerated tissue. The newly formed bone tissues and cells within mandible homotopic grafts were observed to be arranged in a highly organized manner (Figure 1A). The staining was dark and robust in the femoral homotopic grafts, indicating their endochondral ossification (Figure 1C). It is evident that the bone sutures of femoral heterotopic grafts exhibit a higher concentration of proteoglycan and red cartilage tissue in comparison to mandible homotopic grafts, albeit with a lower quantity of cartilage than that observed in femoral homotopic grafts (Figure 1B). Quantify the red cartilage tissue stained with safranin green, and the results show that there is a greater amount of cartilage in femoral homotopic grafts (Figure 1F). Meanwhile, Raman spectroscopy was employed to assess alterations in the mineralized bone and cartilage constituents within the transplanted tissue region. The 1063/958 cm^−1^ ratio served as a spectral indicator for evaluating the relative content of cartilage and bone.^20^ Our findings indicate that femoral heterotopic grafts exhibit reduced levels of mineralization and crystallinity, while displaying increased collagen content in comparison to mandibular homotopic grafts. Despite being placed in a mandibular environment more conducive to intramembranous ossification, the femoral heterotopic graft still maintains its original endochondral ossification pattern, with the newly formed bone containing a certain amount of cartilage matrix that is not significantly different from that observed in femoral homotopic grafts (Figure 1D). Quantitative analysis further revealed a successive decrease in the area ratio of femoral homotopic grafts, femoral heterotopic grafts, and mandible homotopic grafts at 1063/958 cm^−1^, indicating a decline in the relative cartilage content (Figure 1E).

Haematoxylin and eosin staining (Figure S1A–C), Masson staining (Figure S1D–F) and Goldner staining (Figure S1G–I) were used for histological analysis of osteochondral regeneration. The staining results, consistent with Safranin O/fast green staining, revealed a higher abundance of collagen fibres following femoral heterotopic grafting compared to mandible homotopic grafting, yet still lower than that observed in femoral homotopic grafting. After 6 weeks of mandible homotopic grafting, the width of the bone sutures was found to be narrower compared to femoral heterotopic grafting and femoral homotopic grafting, and was enveloped by mineralized bone tissue. Abundant osteoblasts populated the bone trabeculae, indicating enhanced maturation of bones. The demarcation between femoral heterotopic grafting and mineralized bone tissue was distinct, revealing visible bone sutures and immature bones. Furthermore, chondrocytes within the callus displayed disarrayed patterns while inflammatory cells aggregated alongside visible cell fragments. In contrast, femoral homotopic grafting exhibited shallow staining and loose tissue accompanied by the presence of inflammatory cells and cell fragments. Compared to mandible homotopic grafting, femoral heterotopic grafting results in a lower proportion of green mineralized bone while preserving red bone, indicating that the paradigm of endochondral ossification remains intact following femoral heterotopic grafting into the mandibular environment.

### 3.2 The specific Hoxc10 site in the mesodermal callus still exists in the mandible neural crest environment after femoral heterotopic graft

**FIGURE 2:**
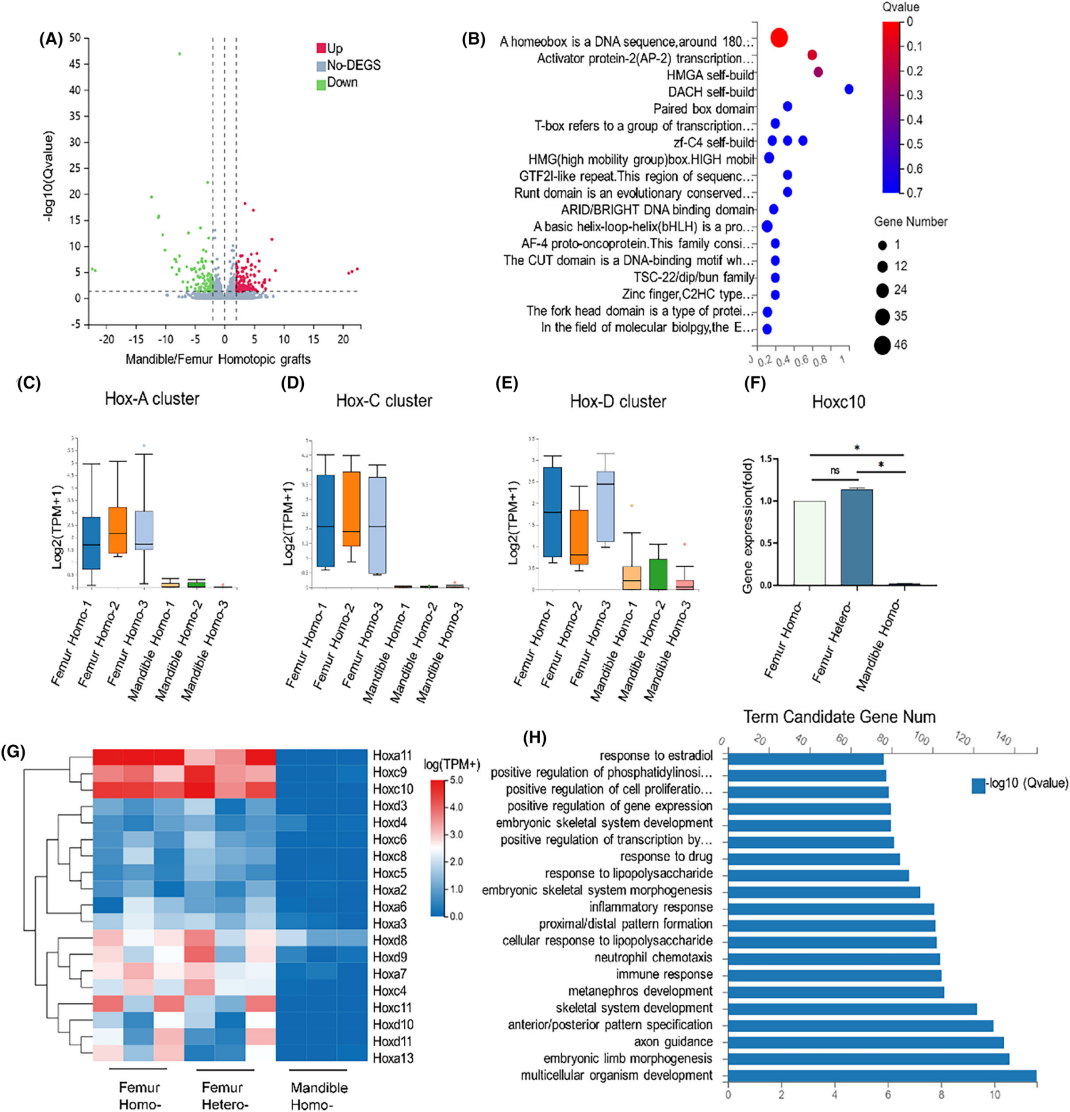
RNA sequencing of callus. (A). Volcano plot of differential gene expression in bone suture tissue after mandible homotopic grafting and femoral homotopic grafting. The genes that were found to be statistically significant (false discovery rate ≤0.05) are highlighted. (B). Transcription factor enrichment analysis of bone suture tissue after mandible homotopic grafting and femoral homotopic grafting. (C–E) were the differential expressions of Hoxa, Hoxc and Hoxd clusters in bone suture tissue after mandible homotopic grafting and femoral homotopic grafting, respectively. (F). qPCR Hoxc10 was used to verify gene expression levels in mandible homotopic grafting, femoral heterotopic grafting and femoral homotopic grafting. (G). Heat maps depicting the expression patterns of distinct Hox family genes across three experimental groups, namely mandible homotopic grafting, femoral heterotopic grafting, and femoral homotopic grafting. (H). Functional enrichment analysis of all Hox genes. The data are presented as the mean ± SD (*n* = 3). *p < 0.05.

To investigate the underlying causes of femoral chondrogenesis and osteogenesis, as well as to determine whether the original gene ecological niche was preserved following femoral heterotopic grafting, bone sutures were collected from SD rats 6 weeks post‐grafting and subjected to RNA sequencing. Prior to this analysis, a comparison of gene expression in the ecological niches of homotopically grafted femurs and mandibles was necessary. Bioinformatics analysis revealed significant differences in gene expression between these two groups, with 1507 differential genes detected 875 up‐regulated and 632 down‐regulated (Figure 2A). Transcription factor enrichment analysis of the femoral homotopic grafting revealed that the Hox gene exhibited the highest intensity of enrichment compared to other transcription factors (Figure 2B). Cell transplantation experiments demonstrated that the expression of the Hox gene played a pivotal role in determining whether transplanted cells successfully integrated into their new location,^21^ thus highlighting its significance as a ‘biological fingerprint’ capable of distinguishing between femurs and mandibles. Given that the femur and mandible originate from mesoderm and neural crest, respectively, we deduced a potential correlation between the distinct healing states following bone grafting and the embryonic origin. Subsequently, functional enrichment analysis was performed on the Hox gene, revealing its pivotal role in intercellular tissue development, regulation of embryonic limb bone morphology, and establishment of the anteroposterior axis during embryogenesis (Figure 2H). Consequently, it is plausible to speculate that apart from governing bone development during embryogenesis, the Hox gene may also exert similar regulatory functions in bone regeneration and healing processes. The Hox gene family is typically categorized into clusters a, b, c and d. However, our sequencing results did not detect any genes belonging to the Hoxb cluster. Consequently, we conducted a comparative analysis of the Hoxa cluster, Hoxc cluster, and Hoxd cluster in femoral homotopic grafting and mandible homotopic grafting. Our findings revealed significant differences in the Hoxc cluster between these two groups (Figure 2C–E). Subsequently, we compared the sequencing results of mandibles homotopic grafting with those of femoral heterotopic grafting and femoral homotopic grafting. Notably, there was a significant disparity in Hoxc10 expression between femoral heterotopic grafting and mandible heterotopic grafting; however, no difference was observed between femoral heterotopic grafting and femoral homotopic grafting. This suggests a state of preserved Hox gene expression (Figure 2G). The qPCR experiment further validated our sequencing results (Figure 2F).

### 3.3 A reservoir of Hoxc10 derived from mesoderm is present within the callus

**FIGURE 3:**
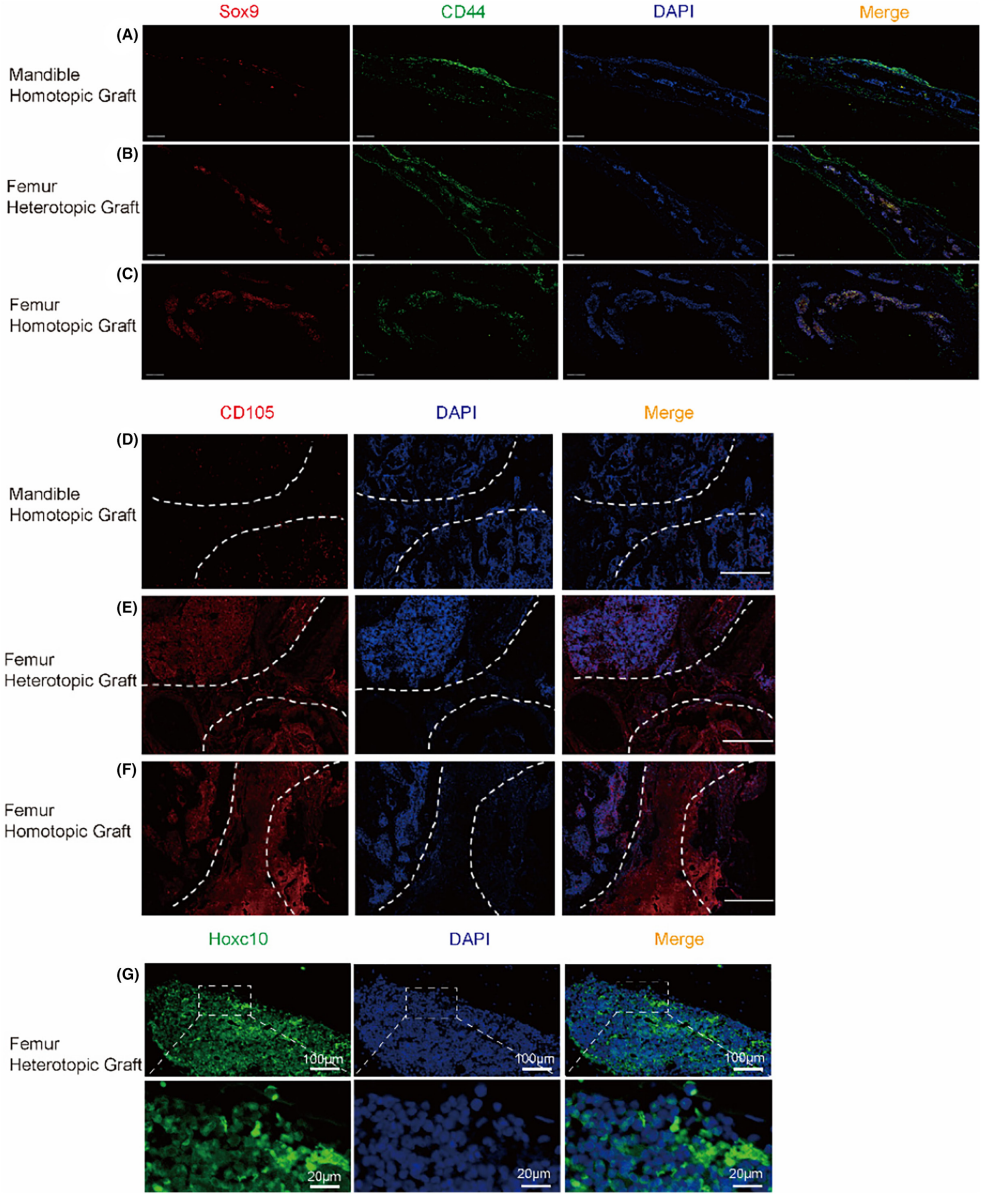
Hoxc10 exists in mesodermal derived callus. The immunofluorescence of Sox9 and CD44 in the mandible homotopic grafting (A), femoral heterotopic grafting (B) and femoral homotopic grafting(C). Sox9 represents cartilage (red), CD44 is a BMSCs marker (green), and DAPI marks the nucleus. (Scale bars, 500 μm) (D–F) represent the immunofluorescence of CD105 in mandible homotopic grafting, femoral heterotopic grafting, and femoral homotopic grafting, respectively. The white dotted line shows the edge of the femoral graft and the mandibular graft, and the middle of the dotted line is the callus. (Scale bars, 200 μm) (G) Localization of Hoxc10 at callus in femoral heterotopic grafting. (Scale bars, 100 μm; Scale bars, 20 μm) The data are presented as the mean ± SD (*n* = 6). *p < 0.05.

Considering that the differentiation direction of BMSCs determines the osteogenic mode of bone, we then concluded that it was the BMSCs that mediated the formation of mandibular cartilage after grafting. To demonstrate this, three sets of bone callus from the graft area were removed for immunofluorescence staining (Figure 3A–C), and the marker CD44 of BMSCs in the callus tissue was found to be co‐located with the chondrogenic related gene Sox9. CD105, another marker of BMSCs, can also be observed in callus tissue (Figure 3D–F). That is to say, after bone defect graft, the differentiation direction of BMSCs is closely related to the mode of bone healing. We speculated that Hoxc10 is related to bone healing and regeneration after graft. So we took the above sequencing results into account and observed the localization of Hoxc10 in the callus (Figure 3D), which suggests that Hoxc10 is involved in bone repair after graft.

### 3.4 The Transwell system emulates the bone niche and exhibits the phenomenon of ‘positional memory’ during bone regeneration

**FIGURE 4:**
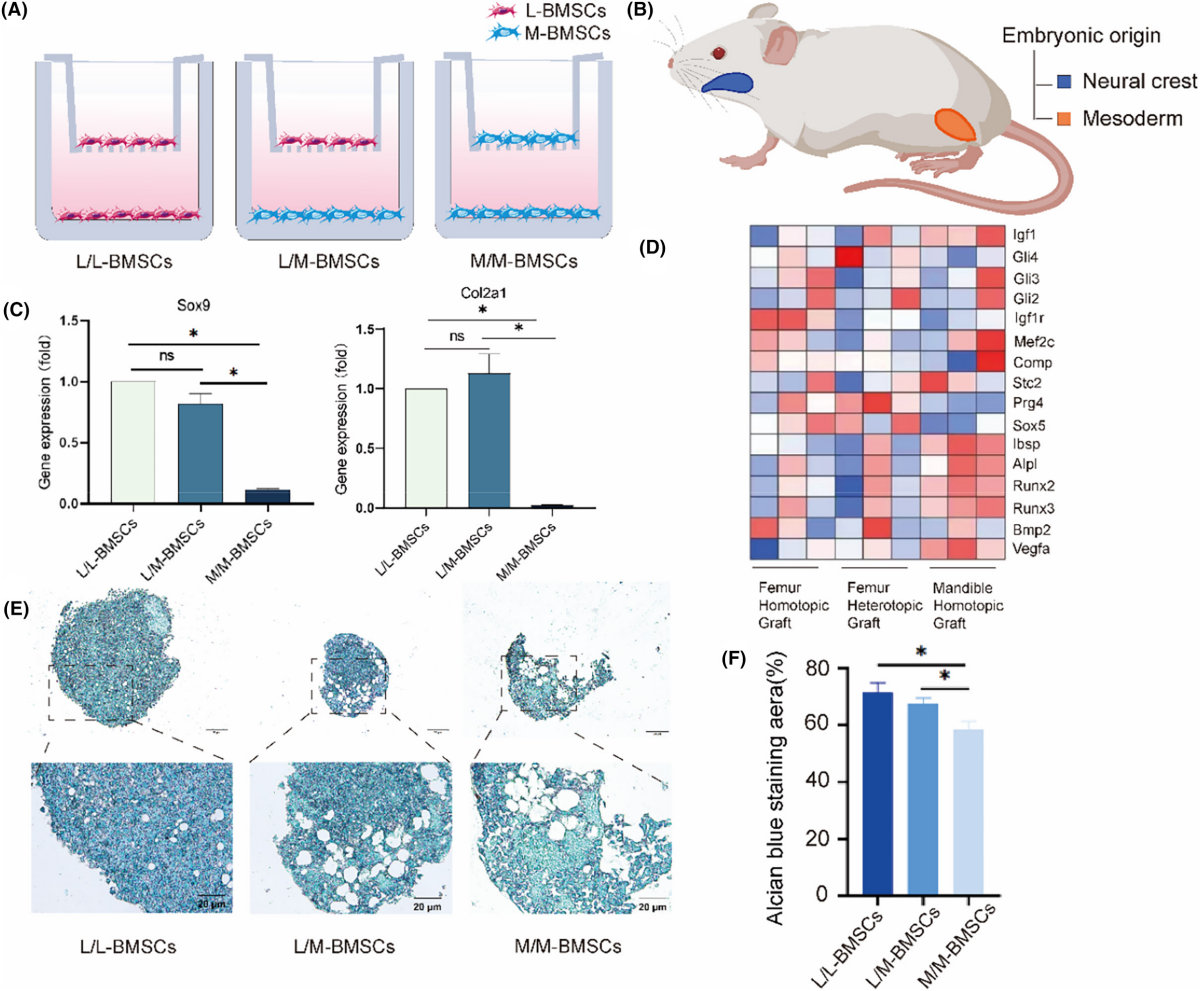
Hoxc10 is retained in L/M‐BMSCs in vitro. (A) Schematic of Transwell co‐culture model of L‐BMSCs and M‐BMSCs. (B) Schematic of the limb bones and mandibles from different embryonic origins. The mandible is of neural crest origin (blue) and the limb bone is of mesodermal origin (orange) (C) qPCR verified the gene expression levels of Sox9 and Col2a1 before and after co‐culture of L‐BMSCs and M‐BMSCs. (D) The proliferation, osteogenic and chondrogenic genes of femoral homotopic grafting, femoral heterotopic grafting and mandibles homotopic grafting. (E) After 21 days of chondrogenic induction in the upper layer cells of Transwell model before and after co cultivation with L‐BMSCs and M‐BMSCs, blue stained proteoglycans were observed using Alcian blue. (F) Quantitative analysis of Alizarin blue staining before and after co culture of L‐BMSCs and M‐BMSCs. The data are presented as the mean ± SD (*n* = 3). *p < 0.05.

In order to verify whether the effect of observation in vivo depends on the interaction between BMSCs of different embryonic origins, we extracted long bone marrow mesenchymal stem cells (L‐BMSCs) and mandibles bone marrow mesenchymal stem cells (M‐BMSCs) from mesoderm derived long bone and neural crest derived mandibles bone respectively in vitro. Flow cytometry and the tri‐differentiation ability of BMSCs proved the successful extraction of primary L‐BMSCs and M‐BMSCs (Figure S2). We used the Transwell model to simulate bone niches in vitro (Figure 4A). The upper layer of Transwell represents the transplanted bone block, while the lower layer represents the environment of the implant bed. As in previous studies, long bones derived from mesoderm, in which Hoxc10 gene is highly expressed, while Hoxc10 is not detected in mandible from neural crest (Figure 4B). We observe whether the differentiation direction of upper long bones BMSCs will change with the presence of lower mandibles BMSCs. Referring to animal experimental results, we also focused on the expression of chondrocyte regulatory factors Sox9 and Col2a1 before and after co‐culture. In vivo experiments, we demonstrated that the amount of cartilage produced by femoral heterotopic grafting was more than that of well healed mandibles homotopic grafting. Therefore, we observed the results of the corresponding two groups, and qPCR analysis confirmed the same results as in vivo: the L/M BMSCs had an increase in cartilage related genes (Figure 4C) compared with the M/M BMSCs. This indicates that the differentiation of BMSCs into cartilage during the osteogenic process leads to poor bone healing after femoral heterotopic grafting. There was no difference in the expression of Sox9 and Col2a between L/L BMSCs and L/M BMSCs (Figure 4C), indicating that the chondrogenic ability of long bones after grafting into the mandibles was preserved. In order to further explore the mode of callus formation after bone grafting, we analysed known cell proliferation and differentiation genes (Figure 4D). For cartilage related marker genes, especially Prg4 and Sox5, L/L BMSCs and L/M BMSCs have similar expression levels and are higher than M/M BMSCs. Collect BMSCs from Transwell upper layer in three groups and induce 3D culture for 14 days, then perform alcian blue staining to verify the deposition of cartilage matrix in BMSCs. Alcian blue results showed that both L/L BMSCs group and L/M BMSCs group showed more blue‐stained proteoglycan at the same time, which proved that it would form cartilage before osteogenesis, while M/M BMSCs group had small and loose chondrospheres with the weakest staining intensity, which proved that its chondrogenic ability was the weakest (Figure 4E). Image J analysis of the percentage of Alcian blue staining area also showed that the L/L BMSCs group had the largest blue staining area and the strongest chondrogenic ability (Figure 4F).

### 3.5 Hoxc10 was correlated with the retention of callus cartilage following graft

**FIGURE 5:**
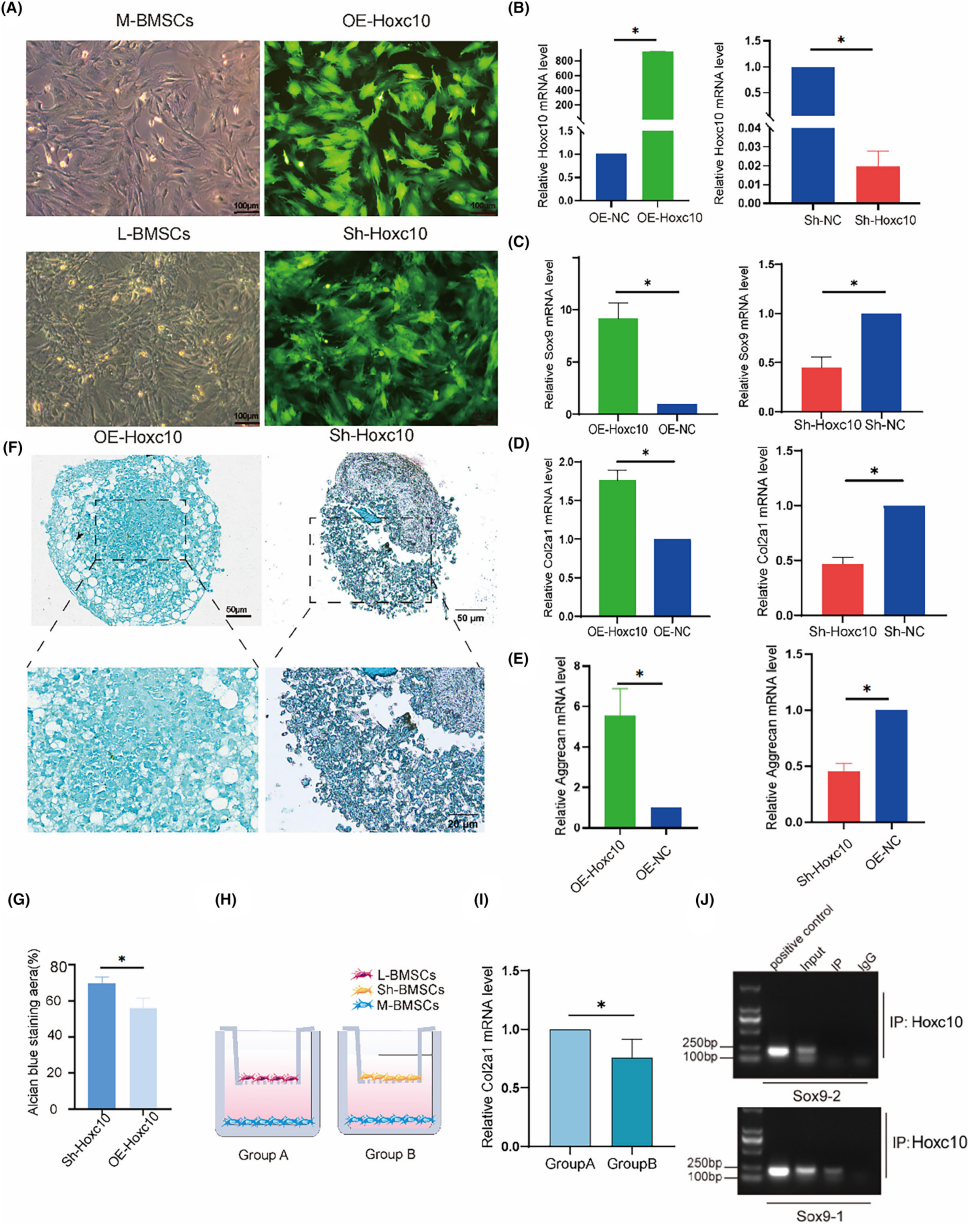
Hoxc10 is positively correlated with cartilage. *n* (C) q‐PCR validated the expression levels of Sox9 gene after overexpression and knockout of Hoxc10. (D) q‐PCR validated the expression levels of the Col2a1 gene after overexpression and knockout of Hoxc10. (E) q‐PCR validated the expression levels of Aggrecan gene after overexpression and knockout of Hoxc10. (F) The proteoglycan of BMSCs after overexpression and knockdown Hoxc10 was observed by Alcian blue staining 21 days after chondrogenic induction. (G) Quantitative analysis of Alcian blue staining. (H) Schematic diagram of co‐culture of L‐BMSCs and M‐BMSCs with and without Hoxc10 knockout. (I) Col2a1 gene expression in L‐BMSCs after Hoxc10 knockout and co‐culture with M‐BMSCs compared to control. (J) ChIP experiment of Sox9 and Hoxc10 protein binding. The data are presented as the mean ± SD (*n* = 3).*p < 0.05.

Subsequently, we wanted to further determine the relationship between differentially expressed Hoxc10 and chondrogenesis of transplanted callus. Based on fluorescence staining, we determined the efficiency of successfully upregulating and downregulating Hoxc10 (Figure 5A). We used qPCR to detect the overexpression and knockout efficiency of Hoxc10 (Figure 5B), as well as the expression of cartilage differentiation markers (Sox9, Col2a1, Aggrecan) in BMSCs after Hoxc10 overexpression and knockout (Figure 5C–E). These results indicate that Hoxc10 is positively correlated with cartilage. Alcian blue staining revealed that overexpression of Hoxc10 resulted in a more compact cartilage structure and nearly complete reconstitution of M‐BMSCs into cartilage‐like tissue, as evidenced by increased proteoglycan staining intensity (Figure 5F). This was also shown by the quantitative analysis of Alcian blue. L‐BMSCs and M‐BMSCs were co‐cultured and named group A, while L‐BMSCs and M‐BMSCs were co cultured and named group B after knocking down Hoxc10 (Figure 5H). It was observed that the chondrogenic ability of group B decreased (Figure 5I). This proves that consuming Hoxc10 may offset the chondrogenic phenomenon caused by Hoxc10's ‘positional memory’ after grafting. To further prove the relationship between Hoxc10 and chondrogenesis, we predicted that the chondrogenesis marker gene Sox9 has two sites that may bind to Hoxc10. ChIP experiments have shown that the Sox9‐1 site has a direct binding with Hoxc10 (Figure 5J).

### 3.6 Hoxc10 deletion facilitates the healing process of heterotopic femoral bone graft

**FIGURE 6:**
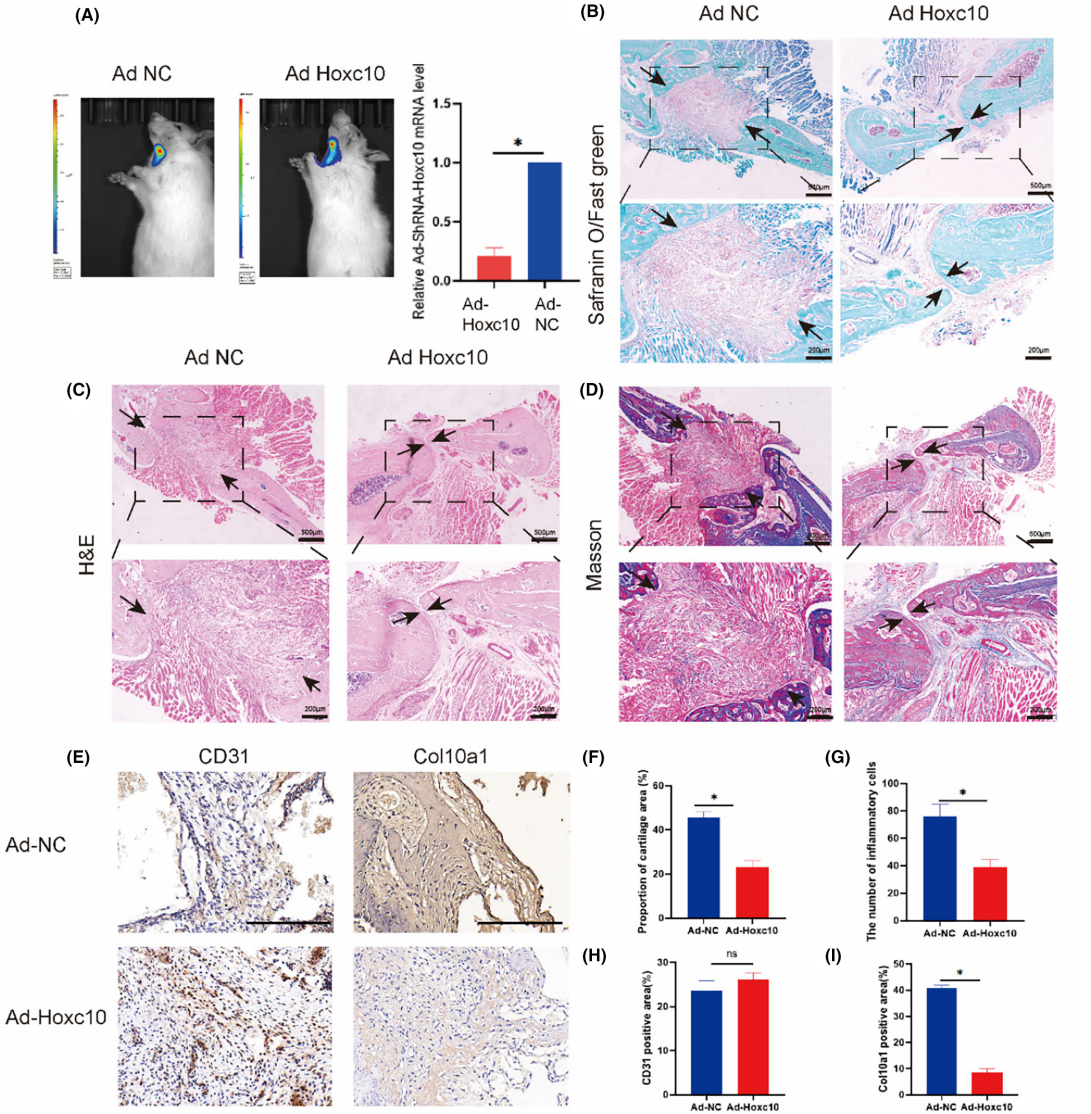
Hoxc10 deletion facilitates the healing process of heterotopic femoral bone graft. (A)The experimental and control groups were imaged on day 4 using an in vivo bioluminescence imaging system. RNA was extracted after Adenovirus (AD)‐Hoxc10‐shRNA virus transfection, and q‐PCR experiments were performed to verify the viral transfection efficiency. Safranin O/fast green staining (B), haematoxylin and eosin staining (C), and Masson staining (D) were used to evaluate the effect of cartilage healing after Hoxc10 knockdown compared with control group. The distance between black arrows represents the width of the suture after healing. (Scale bar = 500 μm at lower magnifications, Scale bar = 200 μm at higher magnifications). (E) Immunohistochemistry to evaluate the expression levels of CD31 and Col10a1 before and after Hoxc10 knockdown. (Scale bar = 500 μm) (F) Quantification of red cartilage tissue stained with Safranin O/fast green staining after Hoxc10 knockdown. (G) Quantitative analysis of inflammatory cells in haematoxylin and eosin staining. (H) Quantification of CD31 before and after Hoxc10 knockdown. (I) Quantification of Col10a1 before and after Hoxc10 knockdown. The data are presented as the mean ± SD. The experiment was repeated three times. (*n* = 3).

Next, in order to determine whether interference with Hoxc10 after femoral heterotopic grafting can change original endochondral ossification mode, we injected adenovirus vector between the femur grafts and the mandibles after femoral heterotopic grafting, and injected empty vector as control. Four days later, the expression of adenovirus was detected by bioluminescence and photographic images, which confirmed that the local injection was successful (Figure 6A). We observed Safranin O/fast green staining (Figure 6B), Masson staining (Figure 6C), and haematoxylin and eosin staining (Figure 6D) in SD rats, and the results showed that the control group bridged the two grafted bone blocks more through muscle fibres, while the adenovirus group bridged the bone matrix more tightly(Figure 6C), the bone tissue structure was relatively complete, arranged compactly, with densely packed and connected bone trabeculae(Figure 6D), and less red cartilage formation compared to the control group(Figure 6B). This means that the femoral bone graft after knocking out Hoxc10 may change its original ‘positional memory’ and the way endochondral ossification. This method can further accelerate the healing time after bone grafting. To evaluate the changes in femoral grafts after adenoviral knockout of Hoxc10, we assessed the angiogenic capacity and chondrocyte hypertrophy indicators. Immunohistochemistry showed that although CD31 did not change significantly after knockout (Figure 6E,G), Col10a1 was significantly reduced (Figure 6E,H), which proves that the loss of Hoxc10 may lead to reduced cartilage formation and may change its mode of chondrogenesis.

## 4 DISCUSSION

While the application of biomaterials in bone regeneration has garnered attention in recent years, autologous bone grafting remains the gold standard for clinical bone repair strategies. Studies have demonstrated that the success of bone graft healing is closely linked to the selection of an appropriate donor site for autologous bone blocks.^22^, ^23^ Consequently, investigating variations in bone tissue across different regions and their impact on bone healing has emerged as a prominent area of interest within biological and medical research. In this study, we present a fascinating discovery that limb bones not only exhibit Hox site specificity but also retain ‘positional memory’ properties when transplanted into a neural crista‐derived mandible environment. This particular attribute renders the limb bones relatively unaffected to a certain degree post‐grafting, thereby preserving their chondrogenic potential and resulting in a diminished efficacy of bone graft repair and regeneration compared to mandibular homotopic grafting. Furthermore, this characteristic is modulated by Hoxc10.

Importantly, the Hox gene's ‘positional memory’ throughout the life cycle ensures the retention of its regional characteristics into adulthood, thereby summarizing embryonic origin through its expression and location distribution.^24^ Notably, this characteristic is also maintained in adult and even older fibroblasts.^24^ Similarly, long‐term passages do not significantly alter the conserved expression level of the Hoxa10 gene in adult satellite cells.^25^ While extensive research has been conducted on the Hox code during embryonic development, it remains unclear whether this same code persists in bone healing regeneration to maintain a state of ‘positional memory’. Leucht's investigation delves into exploring the persistence of Hox gene expression during regeneration. When grafting Hox‐positive expressing femurs into Hox‐negative expressing mandibles, a persistent presence of the Hox gene was observed.^7^ However, the article lacks clarity regarding which cluster of Hox genes plays a crucial role in the healing phenotype following bone grafting, and the osteogenesis mode and mechanism by which Hox genes are retained in the mandible environment remain unclear. While this phenomenon of ‘positional memory’ may contribute to homeostasis, it also imposes limitations on their plasticity and regenerative capacity. In this study, our objective is to expand upon the current understanding of ‘positional memory’ under regenerative conditions to enhance comprehension of this phenomenon and explore its potential application for promoting bone graft healing.

In order to assess the bone healing efficacy and osteogenic mechanism of mesoderm‐derived long bones transplanted into mandibles of neural crest origin, we generated homotopic and heterotopic models involving both mandibular and femoral grafts. Our findings demonstrate successful mandibular homotopic grafting within the designated timeframe, accompanied by minimal cartilage formation. However, when limb bones were transplanted onto mandibular defects, direct bone formation was absent post‐grafting; instead, a cartilaginous corpus callosum or callus was observed. This suggests that femurs primarily utilize cells from their own source for repair while employing their inherent chondrogenic osteogenesis approach following graft. These results are consistent with previous research outcomes by Douarin.^21^

Next, we aimed to investigate whether this phenomenon is mediated by the Hox gene. To address this, we performed RNA sequencing on three distinct groups of bone suture tissues post‐grafting. Our findings revealed a pivotal role for the Hox gene in this process, with particular emphasis on the Hoxc10 gene within the Hoxc cluster. In line with these observations, Rux et al. investigated into osteoblast and cartilage differentiation defects in cells harbouring mutations in Hox genes. Specifically, they demonstrated that loss of function in the Hox11 family can lead to impaired fracture repair characterized by diminished cartilage formation.^26^ Experiments involving the transplantation of embryo cells have demonstrated that Hox gene expression plays a pivotal role in determining the successful integration of transplanted cells into new locations.^27^ Previous studies have predominantly focused on the contribution of Hox genes to tissue homeostasis, such as skin, bones, and muscles.^28^, ^29^, ^30^, ^31^ However, it is precisely due to this stability that these genes lose their plasticity and exhibit detrimental effects during regeneration processes.^32^ Consequently, inhibiting Hox gene expression emerges as a promising strategy for maintaining pluripotency.^33^ The fluorescence co‐localization and co‐culture analyses both demonstrated that following femoral heterotopic grafting, the femoral bone grafts exhibited sustained expression throughout the healing process, thereby retaining their ‘positional memory’ and recapitulating the embryonic expression profile of Hoxc10. Rinn's study revealed that adult fibroblasts can maintain the transcription program of Hoxa13 to preserve ‘positional memory’, while Douarin further substantiated this by demonstrating successful transplantation of mesoderm cells containing specific Hox codes into a neural crest environment.^7^ These findings align closely with our own results.

In the present study, we conducted a series of experiments to investigate the potential role of the Hoxc10 gene in regulating cartilage formation following femoral bone grafting. Our results revealed a positive correlation between Hoxc10 expression levels and cartilage development through both overexpression and knockdown Hoxc10. Furthermore, subsequent animal experiments provided additional evidence supporting the notion that downregulation of Hoxc10 after femoral heterotopic grafting to mandible can reduce cartilage formation after transplantation. However, it is not clear if the deletion of Hoxc10 accelerates the process of endochondral ossification or if it changes the healing mode to intramembranous ossification. Moreover, whether the reduction of cartilage formation ultimately leads to superior clinical outcomes is not obvious. We currently believe that reduced cartilage formation may promote accelerated bone healing, especially in the mode of intramembranous ossification. The process of cartilage transforming into bone is relatively slow, so reducing cartilage formation may mean entering the osteogenic stage directly, thereby shortening the healing time.^34^ But this may not be certain. This also depends on the specific clinical situation and the need for osteogenic mode. For example, for small bone injuries that require rapid repair, the advantage of intramembranous ossification is more pronounced; But for large bone transplants, cartilage formation remains an indispensable part of the repair process. Therefore, in the short term, the reduction of cartilage formation may accelerate bone healing, but in the long run, whether it will bring superior clinical effects requires us to consider the strength, stability, and functional recovery of bones in future experiments to further improve the experimental results. Collectively, these findings suggest that targeting Hoxc10 may hold promise as a therapeutic strategy for improving outcomes in patients with prosthetic mandible defects undergoing autogenous limb bones grafts.

## 5 CONCLUSION

Our experiment demonstrated that after conducting a co‐culture experiment involving L‐BMSCs and M‐BMSCs, the assimilation of Hoxc10 gene was not observed, thereby preserving the positive ecological niche and facilitating differentiation of L‐BMSCs into chondrocytes in the mandible environment. Specifically, we found that the presence of Hoxc10 gene in limb bone grafts promoted cartilage formation, while its deletion expedited fusion between femur limb and mandible. Our study provides valuable insights into the less effective outcomes of autogenous limbs heterotopic grafting compared to homotopic grafting from the mandible. Consequently, it is crucial to consider the molecular characteristics of mesoderm and neural crest bones in order to enhance the success rate of craniomaxillofacial grafting through careful gene expression profiling. However, further research is warranted to directly demonstrate the phenomenon of ‘positional memory’ associated with Hoxc10 and elucidate its specific regulatory mechanism following grafting.

## AUTHOR CONTRIBUTIONS

Conceptualization, X.W.; methodology, H.S., Y.H., X.C., and N.L.; validation, H.S., Y.H., and J.W.; formal analysis & investigation, H.S., J.H., S.X., and X.W.; resources, H.S., Y.H., Q.X.,X.W.; writing—original draft, H.S., X.W; writing—review & editing, Q.X., X.C., N.L., J.W., X.Z., Y.Z. J.H., and S.X.; supervision, X.W.; project administration, X.W.; funding acquisition, X.W.,P.Z., S.X.

## CONFLICT OF INTEREST STATEMENT

The authors declare no competing interests.

## Supporting information


Data S1.
