# Supplementary material for: Hoxc10‐mediated ‘positional memory’ regulates cartilage formation subsequent to femoral heterotopic grafting
Source: J Cell Mol Med. 2024 Oct 21;28(20):e70140. doi: 10.1111/jcmm.70140 (PMC11493555; doi:10.1111/jcmm.70140)
Supplement: Supplementary file 1 — Data S1. [file JCMM-28-e70140-s001.docx]

Supplementary Information


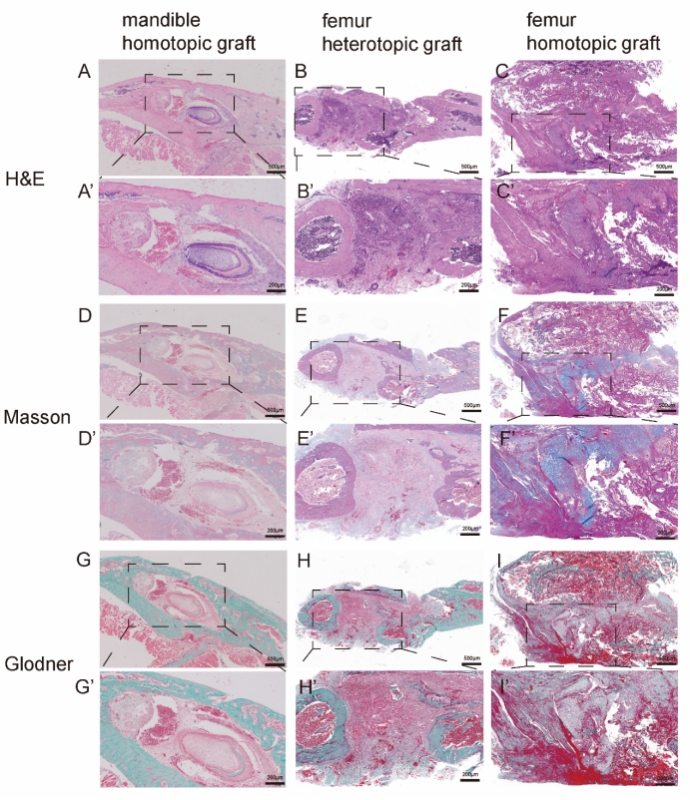


Fig.1 H&E, Masson and Glodner staining of homotopic and heterotopic grafting (n=3). From left to right are, in order, representative images of mandible homotopic graft, femoral heterotopic graft and femoral homotopic graft. H&E staining (A-C), Masson staining (D-E) and Goldner staining (G-I) for three groups of grafting from top to bottom. Scale bar=500μm at lower magnifications, Scale bar=200μm at higher magnifications.


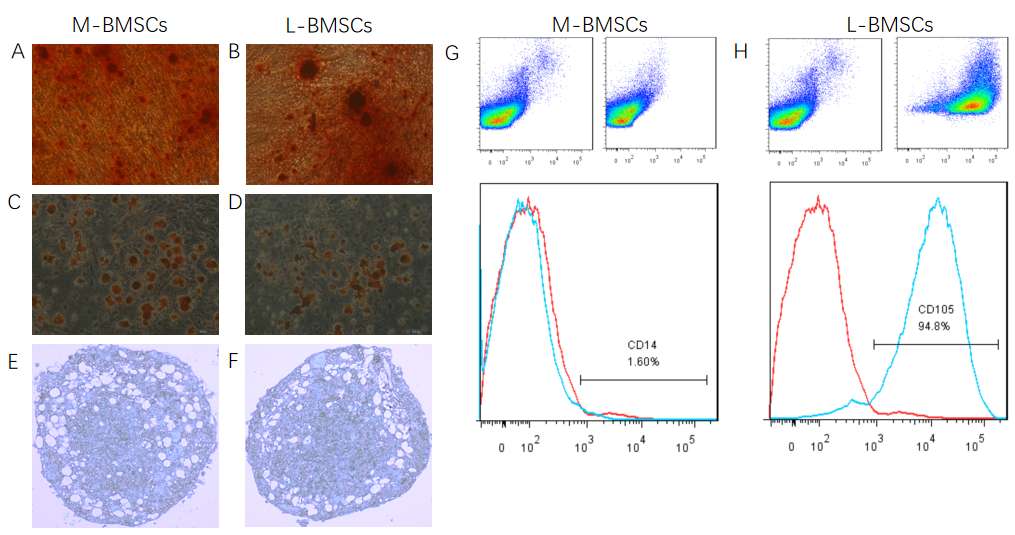


Fig.2 Identification of primary L-BMSCs and M-BMSCs. (A-B) Osteogenic differentiation of primary L-BMSCs and M-BMSCs. (C-D) Adipogenic differentiation of primary L-BMSCs and M-BMSCs. (D-E) Chondrogenesis of primary L-BMSCs and M-BMSCs. (G-H) Flow cytometry using CD105^+^, CD14^-^ antibodies to identify primary L-BMSCs and M-BMSCs.


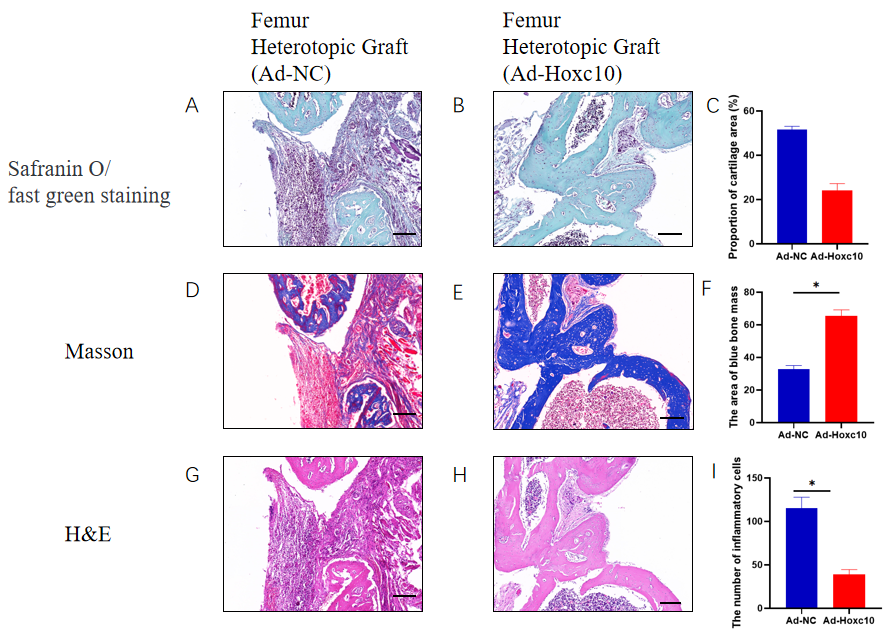


Fig.3 Histological staining of femoral heterotopic grafts after knockout of Hoxc10. (A-B) Safranin O/Fast Green Staining of Ad NC and Ad Hoxc10. (C) Quantification of Safranin O/Fast Green Staining. (D-E) Masson Staining of Ad NC and Ad Hoxc10. (F) Quantification of Masson Staining. (G-H) H&E Staining of Ad NC and Ad Hoxc10. (I) Quantification of H&E staining.Scale bar=200μm.The data are presented as the mean ± SD. The experiment was repeated three times. (n = 3).
